# Prevalence of Anxiety and Depression in Patients with Inflammatory Bowel Disease

**DOI:** 10.1155/2017/6496727

**Published:** 2017-10-18

**Authors:** Glynis Byrne, Greg Rosenfeld, Yvette Leung, Hong Qian, Julia Raudzus, Carlos Nunez, Brian Bressler

**Affiliations:** ^1^Department of Medicine, University of British Columbia, Vancouver, BC, Canada; ^2^Department of Medicine, Division of Gastroenterology, University of British Columbia, Vancouver, BC, Canada; ^3^Centre for Health Evaluation and Outcome Sciences, University of British Columbia, St. Paul's Hospital, Vancouver, BC, Canada; ^4^Department of Psychiatry, University of British Columbia, Vancouver, BC, Canada

## Abstract

**Background:**

Inflammatory bowel disease (IBD) patients are not routinely screened for depression and anxiety despite knowledge of an increased prevalence in people with chronic disease and negative effects on quality of life.

**Methods:**

Prevalence of anxiety and depression was assessed in IBD outpatients through retrospective chart review. The presence of anxiety and/or depression was determined using the Patient Health Questionnaire-9 and Generalized Anxiety Disorder-7 self-report questionnaires or by diagnosis through psychiatric interview. Patient demographics, disease characteristics, and medication information were also collected. Multivariable analysis was used to determine associations between patient factors and depression and anxiety.

**Results:**

327 patient charts were reviewed. Rates of depression and anxiety were found to be 25.8% and 21.2%, with 30.3% of patients suffering from depression and/or anxiety. Disease activity was found to be significantly associated with depression and/or anxiety (*p* = 0.01). Females were more likely to have anxiety (*p* = 0.01).

**Conclusion:**

A significant proportion of IBD patients suffer from depression and/or anxiety. The rates of these mental illnesses would justify screening and referral for psychiatric treatment in clinics treating this population. Patients with active disease are particularly at risk for anxiety and depression.

## 1. Introduction

The prevalence of depression and anxiety is higher in patients with chronic diseases compared to the general population [1] and having a long term medical illness is a risk factor for depression [2]. There is evidence that inflammatory bowel disease (IBD), which includes Crohn's disease (CD) and ulcerative colitis (UC), is associated with higher rates of anxiety and depression compared to the general population [3] and that depression is associated with decreased quality of life in IBD patients [4]. Research on patients with both chronic disease [5] and IBD [6] reveals that the presence of an anxiety or depressive disorder is associated with poor treatment compliance. In addition, depressive disorders are associated with poorer functioning and higher morbidity and mortality in persons with chronic medical conditions [7]. Provision of treatment for mental disorders may improve long term outcomes, and it is therefore important to identify patients at greater risk of anxiety and depression so that they can be offered the appropriate treatment and support.

In a previous Canadian study, IBD cases were extracted from two nationally representative health surveys and depression rates were examined [8]. Depression was diagnosed using a structured diagnostic interview and cases were identified based on self-report of “a bowel disorder such as Crohn's disease or colitis” that has lasted longer than 6 months and been diagnosed by a health professional. The twelve-month depression rates were found to be 14.7% and 16.3% in the two survey samples for IBD patients, rates that are triple those in the general population. Depression rates were found to be higher among those that were female, single, and younger, had greater pain, and had functional limitations. A limitation of this study was that cases were identified through self-report without any medical record verification, and the nonspecific wording of the self-report question may have resulted in inaccurate reporting of IBD.

The primary aim of this study was to determine the prevalence of anxiety and depression in IBD patients attending a gastroenterology outpatient clinic. A secondary aim was identification of patient characteristics associated with increased rates of these mental disorders.

## 2. Methods

Prevalence of anxiety and depression was assessed in IBD outpatients attending a gastroenterology clinic associated with a tertiary care academic hospital (St. Paul's Hospital, Vancouver, Canada) from January to July 2016. All consecutive IBD patients attending the outpatient clinic were asked to fill out the Generalized Anxiety Disorder-7 (GAD-7) and Patient Health Questionnaire-9 (PHQ-9) questionnaires at their medical appointments to screen for anxiety and depression. The GAD-7 and PHQ-9 are brief self-report scales used to identify Generalized Anxiety Disorder and depression, respectively. Validated in the primary care setting, the GAD-7 has a sensitivity of 89% and a specificity of 82% [9], and the PHQ-9 has a sensitivity of 88% and a specificity of 88% [10] when using a cut-off of 10 or greater as a positive result. Patients were offered referral to psychiatry if they screened positive for anxiety and/or depression, if they discussed mood symptoms with the gastroenterologist, or if they requested referral. A psychiatric interview was then conducted and a diagnosis or lack thereof of Generalized Anxiety Disorder (GAD) or major depressive disorder (MDD) was recorded.

Information collected from patient charts included gender, age at time of mental health assessment, smoking status, disease duration, disease diagnosis (UC versus CD), Partial Mayo scores (UC patients), Harvey-Bradshaw Index (CD patients), previous surgical resection, disease phenotype according to the Montreal classification scores, c-reactive protein (CRP, a noninvasive marker of inflammation with a sensitivity of 60.7% and a specificity of 75.9% in IBD patients for endoscopic inflammation using a cut-off >0.5 mg/dL [11]), current medications, and past use of a biologic medication. Information regarding current psychiatric treatment (including pharmacotherapy or psychotherapy), the use of psychiatric drugs including antidepressants and anxiolytic medications, and history of past diagnosis of anxiety or depression were recorded. These variables were not formally assessed in gastroenterology appointments and were therefore only reliably assessed in patients who had appointments with psychiatry. Patients were considered to have anxiety and/or depression given they had a positive score (10 or greater) on the GAD-7 and/or PHQ-9 screening questionnaires and/or a diagnosis of anxiety or depression from psychiatric interview after referral to a psychiatrist. Both screening questionnaire administration and psychiatric interview took place between January 2016 and August 2016. Psychiatry evaluated referred patients for current presence of anxiety or depressive disorders at appointments, and a diagnosis was made based on physician assessment. Active disease was defined as a Partial Mayo score greater than 4 or a Harvey-Bradshaw Index greater than 7, both accepted cut-offs to indicate moderate to severe disease for these two disease activity measures [12, 13].

Univariate and multivariable logistic regression was used to determine if associations existed between patient characteristics and depression and anxiety. The following potential risk factors, which were identified a priori, were investigated for association with mental disorders in IBD patients: gender, disease diagnosis, disease activity, perianal disease, current steroid use, current or previous biologics, current age, and disease duration. The results shown in the current manuscript are from complete case analysis. We recognized the missing data issues in the data set and the analyses based on the complete case might be biased. In order to examine the impact of the missing data on the results of the analyses, a multiple imputation using chained equations was applied as a sensitivity analysis. The imputation process was conducted in SAS and replicated 200 times.

## 3. Results

### 3.1. Study Population

A total of 327 IBD patients were included in the study, Table 1 describes clinical characteristics associated with our cohort. Disease activity is not formally assessed at each clinic visit; therefore, for some patients a Partial Mayo or Harvey-Bradshaw Index score was not provided or possible to calculate for the time of the mental health assessment. Sufficient information to determine disease activity was available for 170/327 patients in the study, 13% had at least moderately active disease using the Partial Mayo score (UC) and Harvey-Bradshaw Index (CD). Sixty-four percent of those with moderately to severely active disease had CRP lab value, 86% of which had a value over 5 mg/L indicating biochemical confirmation of inflammation.

Past psychiatric history and treatment were not formally assessed in gastroenterology appointments; therefore information regarding psychiatric treatment and diagnosis is presented for patients who attended psychiatric appointments only, as these variables were more consistently assessed via psychiatry. Twenty-two patients attended an appointment with psychiatry; of those patients seven were taking an antidepressant medication, three were also taking benzodiazepines, and one patient was receiving cognitive behavioural therapy. Eleven patients had received psychiatric treatment in the past, two had previously been diagnosed with anxiety alone, five with depression alone, and two with anxiety and depression.

### 3.2. Prevalence of Anxiety and Depression

The presence or absence of depression could be assessed for 314 patients, anxiety for 322 patients, and depression and/or anxiety for 317 patients; missing data is due to lack of or incomplete questionnaires. Prevalence with 95% confidence intervals (CI) of anxiety was 21.1% (95% CI: 17.0%–25.9%), 25.5% (95% CI: 21.3%–30.9%) for depression, and 30.3% (95% CI 25.5%–35.6%) for depression and/or anxiety. Of the patients who screened positive for depression and/or anxiety and accepted referral to a psychiatrist, 18 of 20 patients (90%) with positive PHQ-9 scores had a diagnosis of depression confirmed, and 16 of 19 patients (84%) with positive GAD-7 scores had a diagnosis of anxiety confirmed.

### 3.3. Factors Associated with Anxiety and Depression

Univariate analysis found that presence of active disease was significantly associated with depression and/or anxiety (OR: 4.14, *p* value: 0.003), as well as with depression (OR: 4.47, *p* value: 0.002) and anxiety (OR: 3.37, *p* value: 0.02). In multivariable analysis, presence of active disease was independently significantly associated with depression and/or anxiety (OR: 4.28, *p* value: 0.01), as well as with depression (OR: 4.70, *p* = 0.004) and anxiety (OR: 2.97, *p* = 0.05). Male gender was protective from development of anxiety (OR: 0.29, *p* = 0.01). None of the other variables investigated were found to be significantly associated with anxiety or depression. Odds ratios and *p* values from multivariable analysis of each variable with anxiety and/or depression are described in Table 2.

**Figure 1:**
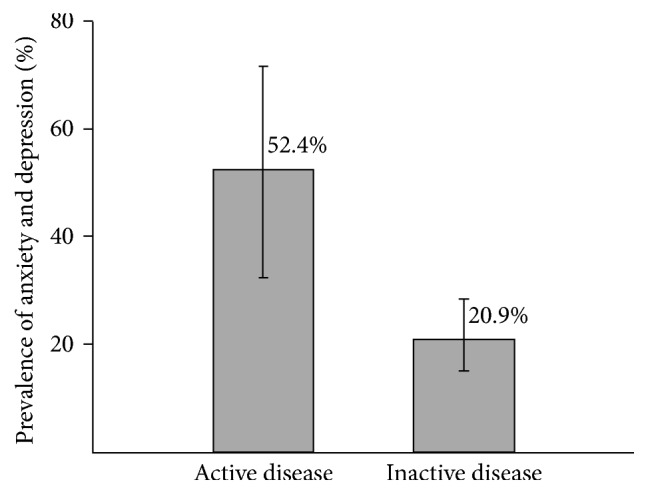
Percentage of patients found to have anxiety and/or depression at present with active and inactive disease. Error bars represent 95% CI.

Disease activity was significantly associated with increased risk of depression and anxiety in IBD patients.  Figure 1 shows the percentage of patients with anxiety and/or depression with active disease and those with inactive disease.

## 4. Discussion

### 4.1. Prevalence of Anxiety and Depression in IBD Patients

The prevalence of anxiety (21.2%) and depression (25.8%) in IBD patients reported in this study is higher than that reported for the general Canadian population. According to the 2013 Statistics Canada Health Survey, the 12-month and lifetime prevalence of depression in the Canadian population were 4.7% and 11.3%, and the 12-month and lifetime prevalence of Generalized Anxiety Disorder were 2.6% and 8.7% [14]. Our results are consistent with findings that patients with long term medical conditions are at increased risk of major depression [2] and with studies that have found the prevalence of anxiety and depression to be higher in IBD patients compared with healthy controls [15–17].

There is variation in the rates of depression and anxiety reported in different studies likely due to differences in populations studied, methods used to assess depression and anxiety, and the period of time within which depression and/or anxiety was assessed (lifetime versus 12 months versus current) [3, 8, 18]. Previous Canadian studies have assessed rates of depression and anxiety in survey samples of self-reported IBD patients [8, 18] and in an IBD patient cohort [19] but none in consecutive patients attending an outpatient clinic. Our study is unique in that it is the first Canadian study to investigate the prevalence of anxiety and depression in consecutive outpatients attending a gastroenterology clinic. Screening for anxiety and depression in consecutive patients may provide a more accurate estimate of the prevalence as this design removes the selection bias introduced when depending on patients to volunteer to be part of a cohort or to reply to a survey. Therefore, our study should provide a more accurate estimate of the point prevalence of anxiety and depression in an outpatient IBD population.

Twelve-month rates of depression reported from two nationally representative surveys with patients identified through self-report of “a bowel disorder such as Crohn's disease or colitis” were 14.7% and 16.3% [8]. Our results show a higher prevalence of depression, likely due to the lack of specificity in the criteria used to diagnose IBD in these survey studies. Another study used survey samples of self-reported IBD patients to assess the prevalence of anxiety and found that IBD patients had 2.18 greater odds of anxiety compared to the general population [18]. Limitations of these studies are that cases of IBD were not verified through medical records, and only 82.6%, 84.7%, and 79.8% of households responded to the surveys, potentially introducing bias due to differences between those who chose to respond and those who did not. It has been observed that those with less education, older age, and greater use of psychopharmaceutical drugs are less likely to participate in survey research [20].

A Canadian cohort study which drew on patients from the University of Manitoba Research Registry found lifetime rates of Generalized Anxiety Disorder (GAD) and major depressive disorder (MDD) in IBD patients to be 13.4% and 27.2% [19]. IBD patients eligible for inclusion in the registry were identified through the database of Manitoba Health. A limitation of this study is that the registry is composed of a subset of IBD patients that may not be representative of all of those living in the community: patients had to be diagnosed within the past 7 years for inclusion and patients needed to agree to participate in the registry, which resulted in only just over half of eligible IBD patients being included. Further, of those eligible in the registry 14% declined to take part in the cohort study and a further 35 of 388 patients initially enrolled were lost throughout the 2-year duration. The characteristics of the patients who agreed and were able to meet the demands of inclusion in the registry and cohort study may vary from those unable or unwilling to participate. Research of nonparticipation in a prospective study on chronic respiratory and cardiovascular diseases found that patients who did not feel well and/or had been admitted to hospital during the last 12 months had lower participation rates [21].

Our study included IBD patients attending a gastroenterology outpatient clinic where all patients are asked to complete the GAD-7 and PHQ-9 questionnaires to limit selection bias. One limitation was that the patient population assessed was drawn from a tertiary IBD clinic; this may result in a patient population with more severe or active IBD, or more comorbidities compared to community samples. Additionally, this study did not include any patients that were currently hospitalized, and because anxiety and depression have been associated with disease flares and inpatient status, prevalence of anxiety and depression may be lower in the population studied compared to inpatient IBD populations [3, 16].

As previously reported in the literature [15, 22], we found that disease activity was significantly associated with increased risk of depression and anxiety in IBD patients. Häuser et al. (2011) [17] found that IBD patients with moderate/severe disease activity had higher rates of depression and anxiety compared to both those with slight disease activity and to an age and sex matched representative sample of the general population. Patients in remission were not at an increased risk of depression and anxiety compared to the general population. We found that prevalence of anxiety and depression did not differ between UC and CD patients, which is consistent with previous research [17]. Female sex was associated with increased risk of anxiety, which has been reported in other studies of patients with IBD [18, 22], as well as in the general population [14, 23].

Variation in methods of psychiatric assessment is likely partially responsible for the variation in reported prevalence of anxiety and depression in IBD patients between studies. Use of screening questionnaires may overestimate rates of depression and anxiety: when patients were assessed using both the DSM-3 criteria and the Hospital Anxiety and Depression Scale (HADS) screening questionnaire rates of psychiatric illness were lower using the DSM criteria [24]. We used the GAD-7 and PHQ-9 screening questionnaires, which have been validated in the primary care setting, to assess anxiety and depression. When using a cut-off of 10 or greater as a positive result for both questionnaires, the GAD-7 has a sensitivity of 89% and a specificity of 82% [9], while the PHQ-9 has a sensitivity of 88% and a specificity of 88% [10]. Some of the symptoms of depression used for screening, such as changes in appetite and fatigue, may overlap with IBD symptoms; therefore, the PHQ-9 may overestimate depression in IBD patients. Assessment of the accuracy of the PHQ-9 screening questionnaire in this population is important. In our study this screening tool seems to be accurate; of those patients who agreed to referral to a psychiatrist, 90% of patients who scored positive on the PHQ-9 had a diagnosis of depression confirmed by psychiatric interview.

The high prevalence of mental disorders we have found in our study is an important problem that requires further attention. A meta-analysis which investigated any correlation between depression and patient compliance in a variety of chronic disease patients (end stage renal disease, angina, cancer, renal transplant, and rheumatoid arthritis) with a variety of interventions (dietary and health behaviour interventions and medications) found that depression was associated with three times greater odds of noncompliance with treatment recommendation [5]. Both depression [25] and presence of psychiatric disorders [6] have been associated with decreased treatment compliance in IBD patients. Adherence to medical treatment in IBD patients is important not only in the short term for preventing flares of disease activity but also to prevent long term complications. Identification and treatment of anxiety and depression in IBD patients may be important in improving treatment adherence and long term patient outcomes. Anxiety and depression in IBD have been shown to contribute to poor health related quality of life (HR-QOL) independent of disease severity [26]. Zhang et al. (2013) [4] examined the role of depression and disease activity as independent factors in predicting HR-QOL. Depression was found to be the most significant predictor of poor HR-QOL in IBD patients, with disease activity being only weakly predictive. Therefore, addressing mental health in patients with IBD may be important in maximizing patient quality of life. Additionally, psychiatric illness in IBD patients has been found to predict high cost outcomes including emergency department visits, IBD-related hospitalizations, and high treatment charges [27]. The potential benefits of mental health treatment, along with the increased prevalence of anxiety and depression in IBD patients, suggest that screening for anxiety and depression in this population would be of value.

## 5. Conclusion

In summary, our study shows that a significant proportion (30.3%) of IBD outpatients suffer from anxiety and/or depression. The prevalence of anxiety and/or depression found in our study justifies screening and referral for psychiatric treatment in clinics treating this population. The finding that disease severity is associated with increased risk of depression and anxiety suggests that those with active disease may benefit from more intense psychiatric screening.

## Figures and Tables

**Table 1:** Characteristics of the patient population.

Variable	Anxiety and/or depression *n* = 96	No anxiety or depression *n* = 231
Age, year (mean, std)	38.57 +/− 13.80	38.78 +/− 14.73
Female sex, *n* (%)	52 (54.2%)	111 (48.1%)
Smoking status, *n* (%)		
Nonsmoker	58 (71.6%)	146 (80.2%)
Past smoker	12 (14.8%)	20 (11.0%)
Current smoker	11 (13.6%)	16 (8.8%)
Disease diagnosis, *n* (%)		
Crohn's disease	38 (39.6%)	86 (37.2%)
Ulcerative colitis	58 (60.4%)	145 (62.8%)
Time since diagnosis, years (mean, std)	11.85 +/− 8.76	10.60 +/− 8.58
Active disease, *n* (%)	11/41 (26.8%)	11/129 (23.4%)
Elevated CRP, *n* (%)	20 (36.4%)	34 (25.9%)
Previous surgical resection, *n* (%)	25 (26.0%)	54 (21.6%)
Perianal disease, *n* (%)	23 (24.0%)	50 (21.6%)
Steroids, *n* (%)	3 (3.2%)	8 (3.5%)
Current/previous biologic, *n* (%)	64 (66.7%)	137 (59.3%)
Depression, *n* (%)	81 (25.8%)	
Anxiety, *n* (%)	68 (21.1%)	
Diagnosis via screening questionnaire, *n* (%)	142 (95%)	
Diagnosis via psychiatric interview, *n* (%)	7 (5%)	
Current psychiatric treatment, *n* (%)	7/22 (31.8%)	
Current psychiatric medication, *n* (%)	7/22 (31.8%)	
Past psychiatric treatment, *n* (%)	11/22 (50%)	
Past diagnosis anxiety/depression, *n* (%)	9/22 (40.9%)	

**Table 2:** Multivariable logistic regression analysis of the association between patient demographic and disease characteristics and the presence of depression and/or anxiety.

Variable	Odds ratio (95% CI)	*p* value
Gender (male)	0.67 (0.31–1.45)	0.31
Disease diagnosis (CD)	0.53 (0.21–1.33)	0.17
Disease activity (active)	4.28 (1.52–12.04)	0.01
Perianal disease	1.28 (0.45–3.62)	0.65
Current steroids	0.73 (0.05–10.6)	0.81
Current or previous biologic use	1.54 (0.62–3.86)	0.35
Current age (year)	0.97 (0.94–1.00)	0.07
Time since diagnosis (year)	1.02 (0.96–1.08)	0.52
